# Long‐term safety and tolerability of lacosamide monotherapy in patients with epilepsy: Results from a multicenter, open‐label trial

**DOI:** 10.1002/epi4.12522

**Published:** 2021-08-02

**Authors:** Elinor Ben‐Menachem, Jacqueline Dominguez, József Szász, Cynthia Beller, Charles Howerton, Lori Jensen, Carrie McClung, Robert Roebling, Björn Steiniger‐Brach

**Affiliations:** ^1^ Institute for Clinical Neuroscience and Physiology, Sahlgrenska Academy University of Gothenburg Gothenburg Sweden; ^2^ Institute for Neurosciences St. Luke’s Medical Center Quezon City Philippines; ^3^ George Emil Palade University of Medicine, Pharmacy, Science and Technology of Târgu Mureş Emergency Clinical County Hospital Targu Mures (Spitalul Clinic Judeţean de Urgenţă Târgu Mureş), Sectia Clinica Neurologie II Târgu Mureş Romania; ^4^ UCB Pharma Raleigh NC USA; ^5^ UCB Pharma Monheim am Rhein Germany; ^6^ UCB Pharma Brussels Belgium

**Keywords:** epilepsy, lacosamide, long‐term, monotherapy, safety, tolerability

## Abstract

The primary objective of this trial (SP1042; NCT02582866) was to assess long‐term safety and tolerability of lacosamide monotherapy (200‐600 mg/day) in adults with focal (partial‐onset) seizures or generalized tonic‐clonic seizures (without clear focal origin). This Phase III, long‐term, open‐label, multicenter, follow‐up trial enrolled patients with epilepsy who were taking lacosamide in, and completed, the previous double‐blind trial (SP0994; NCT01465997). Primary safety outcomes were treatment‐emergent adverse events (TEAEs), discontinuations due to TEAEs, and serious TEAEs. One hundred and six patients were enrolled and received lacosamide: 84 (79.2%) completed the trial and 22 (20.8%) discontinued. The median duration of exposure was 854.0 days, with a median modal dose of 200 mg/day. Ninety‐six (90.6%), 64 (60.4%), and 44 (41.5%) patients had ≥12, ≥24, and ≥36 months of lacosamide exposure, respectively. At least one TEAE was reported by 61 (57.5%) patients. The most common (≥4%) TEAEs were headache (10 [9.4%]), nasopharyngitis (eight [7.5%]), and back pain (five [4.7%]). One (0.9%) patient discontinued due to a TEAE (sudden unexpected death in epilepsy; not considered drug‐related), 14 (13.2%) patients reported serious TEAEs, and seven (6.6%) patients reported TEAEs that were considered drug‐related. Overall, long‐term lacosamide monotherapy was generally well tolerated up to 600 mg/day, with no new safety signals identified.

## 1 INTRODUCTION

Lacosamide is an antiseizure medication (ASM) that selectively enhances slow inactivation of neuronal voltage‐gated sodium channels.^1^ Some second‐generation ASMs (eg, lacosamide) have shown advantages in tolerability and safety versus first‐generation ASMs (eg, controlled‐release carbamazepine).^2^, ^3^ Lacosamide is indicated for treatment of focal (partial‐onset) seizures in patients ≥4 years of age in the United States and European Union,^4^, ^5^ and elsewhere globally. Lacosamide is also indicated as adjunctive therapy for treatment of primary generalized tonic‐clonic seizures in patients (≥4 years) in the United States, European Union, Australia, and Japan.^4^, ^5^, ^6^, ^7^ The maximum recommended dose of lacosamide monotherapy is 400 mg/day in the United States and 600 mg/day in the European Union.^4^, ^5^

Results of a large‐scale, double‐blind trial (SP0993; NCT01243177)^2^ demonstrated that lacosamide was well tolerated as first‐line monotherapy in patients ≥16 years with recently or newly diagnosed focal (partial‐onset) seizures (simple partial, complex partial, or partial evolving to secondarily generalized with clear focal origin) or generalized tonic‐clonic seizures (without clear focal origin), and was noninferior to controlled‐release carbamazepine. The results of a double‐blind extension of SP0993 (SP0994 [NCT01465997]^8^; with the same target lacosamide doses as SP0993 of 200, 400, and 600 mg/day), and post hoc analyses of pooled long‐term efficacy and safety data from SP0993 and SP0994, showed that treatment with lacosamide monotherapy was well tolerated and efficacy was maintained over a median of approximately two years’ treatment.^8^

Open‐label, follow‐up trials provide valuable information regarding the long‐term tolerability and efficacy of ASMs.^9^, ^10^, ^11^ This open‐label, follow‐up trial (SP1042) enabled patients taking lacosamide in SP0994 to continue lacosamide monotherapy for up to three years, or until lacosamide was approved and commercially available for monotherapy in the patient's country. The primary objective of this trial was to assess the long‐term safety and tolerability of lacosamide monotherapy (200‐600 mg/day) in patients with focal seizures or generalized tonic‐clonic seizures (without clear focal origin).

## 2 METHODS

### 2.1 Trial design

SP1042 (NCT02582866) was a Phase III, long‐term, open‐label, multicenter (Europe, North America, Asia Pacific), follow‐up trial that enrolled patients with epilepsy who were taking lacosamide in, and completed, the previous trial (SP0994). Patient informed consent, protocol, and amendments were reviewed by a regional, national, or Institutional Review Board or Independent Ethics Committee. All patients provided written informed consent before enrollment. The trial was conducted in accordance with the International Council for Harmonization Good Clinical Practice and the Declaration of Helsinki.

### 2.2 Patient eligibility

Patients could enroll in this long‐term follow‐up, open‐label trial if they received lacosamide and completed the termination visit in SP0994. Patients not wishing to continue lacosamide therapy after unblinding of SP0994 were tapered off lacosamide and did not enroll in SP1042.

Patients were excluded if they reported a seizure at the third target lacosamide dose (600 mg/day) in SP0994; received investigational drugs or experimental devices in addition to lacosamide; or had a serious adverse event that was ongoing from SP0994. Patients were excluded or discontinued from SP1042 if they required another ASM for any reason, such as seizure control; the patient may have had the additional ASM added before discontinuation.

Women were excluded who were of childbearing potential, pregnant, or breastfeeding and not using effective contraception, unless sexually abstinent for the trial duration.

### 2.3 Lacosamide dosing

Visit 1 of this open‐label trial was the same as the termination visit of SP0994. Clinic visits were scheduled approximately every 26 weeks relative to Visit 1. In SP0994, patients received a dose of lacosamide 200, 300, 400, 500, or 600 mg/day and continued to receive the same dose in SP1042 until further dose adjustments were required. Lacosamide was administered orally twice daily, in two equal doses. During trial visits, the investigator may have maintained the patient's lacosamide dose, or decreased or increased the dose to optimize seizure control and tolerability (by 100 mg/day/week to a minimum of 200 mg/day or a maximum of 600 mg/day).

The maximum trial duration was 164 weeks, including up to a 156‐week treatment period duration and up to an 8‐week end of study period. The end of study period was the period between the end of the treatment period and final visit, during which any patients stopping lacosamide were tapered off at recommended decreasing steps of 200 mg/day/week over a maximum of 6 weeks; a slower taper (eg, 100 mg/day/week) or faster taper was permitted, if medically necessary. Benzodiazepines were permitted as concomitant medications if taken at a maximum frequency of once per week as rescue therapy for epilepsy, or used for nonepileptic conditions.

### 2.4 Safety outcomes

Primary safety outcomes were treatment‐emergent adverse events (TEAEs) observed by the investigator through open‐ended patient interviews and laboratory monitoring, or reported by the patient and/or caregiver, discontinuations due to TEAEs, and serious TEAEs. TEAEs were adverse events that started on or after the first dose of lacosamide in SP1042, or adverse events with worsened intensity on or after the first dose of lacosamide. Based on general safety considerations and safety data from clinical trials with lacosamide, additional TEAEs termed “other significant TEAEs” were given special consideration (Appendix S1).

### 2.5 Statistical analysis

Analyses were conducted on the Safety Set (SS; all enrolled patients receiving at least one lacosamide dose). Due to the observational nature of this open‐label trial, no formal statistical testing was conducted, and outcomes were analyzed using descriptive statistics.

## 3 RESULTS

### 3.1 Patient disposition and baseline characteristics

This open‐label trial was conducted between January 2016 and January 2020; 106 patients were enrolled and received lacosamide (SS; 86 from Europe, one from North America, 19 from Asia Pacific): 84 (79.2%) completed the trial and 22 (20.8%) discontinued. Reasons for discontinuation were consent withdrawn (seven [6.6%]), lost to follow‐up (two [1.9%]), lack of efficacy (two [1.9%]), adverse event (one [0.9%]), and other (10 [9.4%]).

The mean age of patients in the SS was 43.5 years (standard deviation 17.1 years; range 18‐88 years); 58 (54.7%) patients were male. Five (4.7%) patients had concomitant ASMs; three (2.8%) patients had levetiracetam, two (1.9%) carbamazepine, and one (0.9%) valproic acid (one patient had carbamazepine and levetiracetam); as the addition of the ASM before discontinuation was not classified as a protocol deviation, these patients were not excluded from the analyses.

### 3.2 Lacosamide dosing and exposure

The total lacosamide exposure was 229.3 patient‐years (Table 1). The median duration of exposure was 854.0 days, with a median modal dose of 200 mg/day. Ninety‐six (90.6%), 64 (60.4%), and 44 (41.5%) patients had ≥12, ≥24, and ≥36 months of lacosamide exposure, respectively.

**TABLE 1:** Duration of exposure and patient‐years during the treatment period by modal dose (SS)

	Lacosamide modal dose category							
	≤100 mg/day (n = 1)	>100 to ≤200 mg/day (n = 61)	>200 to ≤300 mg/day (n = 4)	>300 to ≤400 mg/day (n = 28)	>400 to ≤500 mg/day (n = 0)	>500 to ≤600 mg/day (n = 12)	>600 mg/day (n = 0)	Any dose (N = 106)
Duration of exposure (days)								
Mean (SD)	520.0 (NE)	804.2 (311.2)	678.3 (397.5)	756.7 (330.3)	‐	856.4 (329.8)	‐	790.1 (318.4)
Median	520.0	868.0	708.0	836.0	‐	973.5	‐	854.0
Min, max	520, 520	91, 1135	191, 1106	139, 1134	‐	58, 1126	‐	58, 1135
Patient‐years exposed	1.4	134.3	7.4	58.0	0	28.1	0	229.3

Abbreviations: NE, not evaluable; SD, standard deviation; SS, Safety Set.

*Note:* Treatment duration (days) was calculated as the date of last dose of lacosamide during the treatment period minus the date of first dose of lacosamide during the treatment period plus one day. If the date of last dose was unknown, the imputed date of last dose was applied. Patient‐years of exposure was calculated as the total exposure in days divided by 365.25.

### 3.3 Safety and tolerability

#### 3.3.1 All TEAEs during the treatment period of SP1042

Sixty‐one (57.5%) patients reported ≥1 TEAE (Table 2). The most frequently reported (≥4%) TEAEs by preferred term were headache (9.4%), nasopharyngitis (7.5%), and back pain (4.7%). Seven (6.6%) patients reported TEAEs considered drug‐related by the investigator. Preferred terms were nausea (two [1.9%] patients), eosinophil count increased, dizziness, epilepsy (reported term: epileptic seizure, as described below), peripheral sensorimotor neuropathy, and restlessness (one [0.9%] patient each).

**TABLE 2:** Incidence of TEAEs during the treatment period (SS)

Patients, n (%)	Lacosamide overall (N = 106)
Any TEAEs	61 (57.5)
Drug‐related TEAEs^a^	7 (6.6)
Serious TEAEs	14 (13.2)
Severe TEAEs	10 (9.4)
Discontinuations due to TEAEs	1 (0.9)
Deaths	1 (0.9)
TEAEs^b^ occurring in ≥2% of all patients	
Headache	10 (9.4)
Nasopharyngitis	8 (7.5)
Back pain	5 (4.7)
Arthralgia	4 (3.8)
Cough	4 (3.8)
Diarrhea	4 (3.8)
Hypertension	4 (3.8)
Cataract	3 (2.8)
Influenza	3 (2.8)
Musculoskeletal pain	3 (2.8)
Osteoarthritis	3 (2.8)
Upper abdominal pain	3 (2.8)
Upper respiratory tract infection	3 (2.8)
Other significant TEAEs^b^	
Atrial flutter	1 (0.9)
Sinus bradycardia	1 (0.9)
Ventricular tachycardia	1 (0.9)

Abbreviations: SS, Safety Set; TEAE, treatment‐emergent adverse event.

^a^ Drug‐related TEAEs were those with a relationship of “related” or those with missing responses.

^b^ Preferred Term (Medical Dictionary for Regulatory Activities, version 16.1).

Most TEAEs had an intensity of mild or moderate, reported by 20 (18.9%) and 31 (29.2%) patients, respectively. Ten (9.4%) patients reported severe TEAEs. Fourteen (13.2%) patients reported serious TEAEs. The only serious TEAEs reported by ≥1 patient by preferred term were osteoarthritis (two [1.9%] patients; severe; not considered drug‐related).

The seizure‐related TEAE of epilepsy (preferred term) was reported for two (1.9%) patients. One of these TEAEs of epilepsy (reported term of epileptic seizure as described above) was mild in intensity and considered drug‐related and not serious, whereas the other TEAE of epilepsy (reported term of one epileptic seizure) was moderate in intensity and considered serious (patient hospitalized) and not drug‐related; in both events, the TEAE resolved and neither led to discontinuation.

One (0.9%) patient taking lacosamide 600 mg/day died (sudden unexpected death in epilepsy; not considered lacosamide‐related by the investigator, and an autopsy was not performed) after approximately 22 months’ treatment; this was the only TEAE leading to discontinuation during the treatment period. This patient also reported a serious TEAE of peripheral sensorimotor neuropathy considered to be severe, drug‐related, and was ongoing at time of death.

#### 3.3.2 Other significant TEAEs

During the treatment period, a total of three “other significant TEAEs” were reported, by three (2.8%) patients (Table 2). All of these events were cardiac‐related and not considered drug‐related. The TEAEs of atrial flutter and ventricular tachycardia were severe and moderate in intensity, respectively; both were considered serious and resolved. The TEAE of sinus bradycardia was ongoing from the beginning of the trial, moderate in intensity, not serious, and not resolved by the end of the trial. These patients were all taking 200 mg/day lacosamide at the time of onset of the TEAE; for each patient, treatment with lacosamide continued and the dose was not changed.

#### 3.3.3 Pregnancies

One patient taking lacosamide 200 mg/day as monotherapy had a positive pregnancy test and stopped taking lacosamide at seven weeks age of gestation. Following birth, there were no indications of failure to thrive, developmental delay, or congenital abnormality. One patient taking lacosamide 400 mg/day as monotherapy had a positive pregnancy test and stopped taking lacosamide at 17 weeks age of gestation. On delivery day, the patient had generalized tonic‐clonic seizures with loss of consciousness. A healthy infant was delivered prematurely with no gross anomalies.

## 4 DISCUSSION

In this open‐label, long‐term trial, lacosamide monotherapy (200‐600 mg/day) was generally well tolerated in adults with focal seizures or generalized tonic‐clonic seizures (without clear focal origin), with no new safety signals. Patients received lacosamide for up to three years, with 90.6% of patients treated for at least one year and 60.4% for at least two years.

The overall percentage of patients reporting any TEAEs during the treatment period in SP1042 was lower in this open‐label trial (57.5%) versus the double‐blind (SP0993; 74%)^2^ and double‐blind extension (SP0994; 64.9%)^8^ trials. In this trial, 0.9% of patients discontinued due to TEAEs and 13.2% of patients reported serious TEAEs, which is lower or slightly higher, respectively, to rates observed in the double‐blind trials.^2^, ^8^ This may reflect an enriched population in the open‐label trial because patients who did not respond to lacosamide in the double‐blind trials, or who reported TEAEs leading to discontinuation, were not enrolled. Additionally, as is typically seen with other ASMs, TEAEs generally occur more frequently during titration of treatment with lacosamide.^12^

In this trial, TEAEs of headache and nasopharyngitis were the most common, similar to results of previous trials of lacosamide monotherapy.^2^, ^8^, ^13^, ^14^ The low doses of lacosamide (median modal dose of 200 mg/day) used in this trial may explain the very low rates of dizziness and fatigue reported (<2% of all patients each), and why no cognitive disorders were reported. There were three “other significant TEAEs” that were all cardiac‐related. While the United States Prescribing Information for lacosamide lists “cardiac rhythm and conduction abnormalities” as an adverse reaction,^5^ the three cardiac‐related events reported in this trial were not considered drug‐related by the investigator. An important safety aspect of ASM exposure throughout pregnancy is risk of adverse effects on fetal growth and neurodevelopment.^15^ In this trial, there were good outcomes with no reported abnormalities for infants born to two patients taking lacosamide monotherapy up to 17 weeks age of gestation. During the treatment period, there were no TEAEs related to cognitive disorder, amnesia, or memory impairment, and no psychotic disorders were reported.

### 4.1 Limitations

This was an open‐label, uncontrolled trial design with no comparator group. However, this trial collected valuable data on the long‐term tolerability of lacosamide monotherapy and enabled patients to continue lacosamide monotherapy for up to three years or until lacosamide was commercially available as monotherapy in their country. Considering the recommended maintenance dose of lacosamide monotherapy is 300‐400 mg/day in the United States,^5^ lacosamide was generally given at a low dose in this trial, with a median modal dose of 200 mg/day. Due to the small sample size in this trial, rare, serious, drug‐related TEAEs may not have been detected.

## 5 CLINICAL RELEVANCE

Overall, long‐term treatment with lacosamide monotherapy was generally well tolerated at doses up to 600 mg/day (with a median modal dose of 200 mg/day) in adults with focal seizures or generalized tonic‐clonic seizures (without clear focal origin) who completed SP0994. The overall safety profile observed is consistent with the currently known safety profile of lacosamide, with no new safety signals identified.

## DISCLOSURE

Elinor Ben‐Menachem has served as a paid consultant for Arvelle Therapeutics, GW Pharmaceuticals, and UCB Pharma. Jacqueline Dominguez received honorarium as a speaker for Hi‐Eisai Pharmaceutical and A. Menarini Philippines. József Szász has received speaking honoraria from and served as a paid consultant for AbbVie, Boehringer Ingelheim, GlaxoSmithKline, Lundbeck, Novartis, Pfizer, Teva, and UCB Pharma. Cynthia Beller, Charles Howerton, Lori Jensen, Carrie McClung, Robert Roebling, and Björn Steiniger‐Brach are employees of UCB Pharma. We confirm that we have read the Journal's position on issues involved in ethical publication and affirm that this report is consistent with those guidelines.

## Supporting information

Supplementary Material
