# Supplementary material for: Long‐term safety and tolerability of lacosamide monotherapy in patients with epilepsy: Results from a multicenter, open‐label trial
Source: Epilepsia Open. 2021 Aug 2;6(3):618–23. doi: 10.1002/epi4.12522 (PMC8408586; doi:10.1002/epi4.12522)
Supplement: Supplementary file 1 — Supplementary Material [file EPI4-6-618-s001.docx]

# SUPPORTING INFORMATION

**Appendix S1**

**TEAEs termed “other significant TEAEs” defined by Medical Dictionary for Regulatory Activities (Version 16.1) Preferred Term:**

- Hepatotoxicity-related terms: hepatitis toxic, hepatotoxicity
- Cardiac- and ECG-related terms: atrial fibrillation, atrial flutter, ventricular fibrillation, ventricular tachycardia, atrioventricular (AV) block second degree, AV block complete, bradycardia, bradyarrhythmia, sinus bradycardia, heart rate (HR) decreased, sick sinus syndrome, cardiac pacemaker insertion
  - All cases of bradycardia, bradyarrhythmia, sinus bradycardia, and HR decreased with reported reduced HR were reviewed and only cases with marked bradycardia (marked reduction in HR) with HR <45 beats per minute were listed as “other significant TEAEs”
- Suicidality-related terms: completed suicide, depression suicidal, suicidal behavior, suicidal ideation, suicide attempt, intentional self-injury, self-injurious behavior, self-injurious ideation, intentional overdose, poisoning deliberate
- Additional terms: syncope, loss of consciousness

**Appendix S2**

**Co-investigator appendix**

The authors acknowledge the SP1042 trial investigators for their contributions to data acquisition: Pamela Agazzi, MD (Ente Ospedaliero Cantonale [EOC], Ospedale Regionale di Lug, Lugano, Switzerland); Petra Baum, MD (Universitätsklinikum Leipzig, Leipzig, Germany); Jörg Berrouschot, MD (Klinikum Altenburger Land, Altenburg, Germany); Silke Biethahn, MD (Kantonsspital Aarau AG, Aarau, Switzerland); Enver Bogdanov, MD (SBEI HPE “Kazan State Medical University” of MoH of RF, Kazan, Russia); Hristina Chilingirova-Ignatieva, MD (Multiprofile Hospital for Active Treatment Pazardzhik, Pazardzhik, Bulgaria); Yong Won Cho, MD (Keimyung University Dongsan Hospital, Daegu, South Korea); Roman Chwedorowicz, MD (Instytut Medycyny Wsi im, Witolda Chodzki, Lublin, Poland); Liliana Cucos, MD (Spitalul Clinic de Urgenţă “Prof. Dr. Nicolae Oblu” Iasi, Iasi, Romania); Anna Czlonkowska, MD (Instytut Psychiatrii i Neurologii, Warsaw, Poland); Filippo Donati, MD (Spitalzentrum Biel AG, Biel, Switzerland); Boris Doronin, MD (LLC City Neurological Center “Sibneuromed”, Novosibirsk, Russia); Andriy Dubenko, MD (KDU “Inst nevr, psyh ta nark AMNU”, Kharkiv, Ukraine); Yngve Hallström, MD (Neuro Center, Stockholm, Sweden); Seung Bong Hong, MD (Samsung Medical Center, Seoul, South Korea); Berit Helena Jonsson Gauffin, MD (Universitetssjukhuset i Linköping, Linköping, Sweden); Larisa Kadina, MD (Likarnia No. 4 Chernihivskoi miskoi rady, Chernihiv, Ukraine); Vladimir Kalinin, MD (Moscow Research Institute of Psychiatry, Moscow, Russia); Reetta Kälviäinen, MD (Kuopio University Hospital, Kuopio, Finland); Kenichi Kashihara, MD (Okayama Kyokuto Hospital, Okayama, Japan); Sasho Kastrev, MD (MHAT “Puls”, Blagoevgrad, Bulgaria); Junya Kawada, MD (Shonan Kamakura General Hospital, Kanagawa, Japan); Christoph Kellinghaus, MD (Klinikum Osnabrück, Osnabrück, Germany); Jae-Moon Kim, MD (Chungnam National University Hospital, Daejeon, South Korea); Mikhail Kissin, MD (State Educational Institution of Higher Professional Education “Saint Petersburg State Medical University I.P. Pavlov”, St. Petersburg, Russia); Susanne Knake, MD (Universitätsklinikum Gießen und Marburg GmbH, Marburg, Germany); Tobias Kniess, MD (Neurologische Klinik Bad Neustadt an der Saale, Bad Neustadt an der Saale, Germany); Yuichi Kubota, MD (TMG Asaka Medical Center, Asaka, Japan); Sang Kun Lee, MD (Seoul National University Hospital, Seoul, South Korea); Liudmila Lipatova, MD (V.M. Bekhterev Psychoneurological Research Institute, St. Petersburg, Russia); Inara Logina, MD (Pauls Stradiņš Clinical University Hospital, Riga, Latvia); Louis-Georges Maillard, MD (CHU de Nancy - Hôpital Central, Nancy, France); Ivan Milanov, MD (Multiprofile Hospital for Active Treatment in Neurology and Psychiatry “Sv. Naum”, Sofia, Bulgaria); Ioana Mindruta, MD (Sapiens Medical Center, Bucharest, Romania); Masahiro Mizobuchi, MD (Nakamura Memorial Hospital, Sapporo, Japan); Hisashi Okada, MD (Nagoya Medical Center, Nagoya, Japan); Tarja Puumala, MD (Helsinki University Central Hospital, Helsinki, Finland); Ildefonso Rodriguez Leyva, MD (Hospital Central Dr. Ignacio Morones Prieto, San Luis Potosi, Mexico); Artemio Roxas, MD (The Medical City, Pasig, Philippines); Lech Szczechowski, MD (Niepubliczny Zakład Opieki Zdrowotnej Wielospecjalistyczna Poradnia Lekarska Synapsis, Katowice, Poland); Shigeya Tanaka, MD (Tanaka Neurosurgical Clinic, Kagoshima, Japan); Anatolii Voloshchuk, MD (Odessa Regional Medical Center of Mental Health, Odessa, Ukraine); Eduard Yakupov, MD (LLC Scientific Research Medical Complex “Your Health”, Kazan, Russia).
